# Impacto da escleroterapia com espuma de polidocanol guiada por ultrassom em pacientes com úlcera venosa

**DOI:** 10.1590/1677-5449.002717

**Published:** 2017

**Authors:** Melissa Andreia de Moraes Silva, Álefy Zanelato Pereira Araujo, Jéssica Funchal do Amaral, Seleno Glauber de Jesus-Silva, Rodolfo Souza Cardoso, Fausto Miranda

**Affiliations:** 1 Faculdade de Medicina de Itajubá – FMIt, Cirurgia Vascular, Itajubá, MG, Brasil.; 2 Universidade Federal de São Paulo – UNIFESP, Escola Paulista de Medicina, Cirurgia Vascular, São Paulo, SP, Brasil.

**Keywords:** escleroterapia, úlcera varicosa, cicatrização, recidiva

## Abstract

**Contexto:**

A escleroterapia com espuma de polidocanol guiada por ultrassom tem sido utilizada no tratamento de pacientes com úlceras venosas. É um procedimento minimamente invasivo e de fácil execução, porém apresenta taxas de recidiva elevadas.

**Objetivos:**

Relatar a evolução a curto e médio prazo de pacientes com úlcera venosa tratados com escleroterapia com espuma de polidocanol guiada por ultrassom.

**Métodos:**

Foram reavaliados 19 pacientes submetidos ao tratamento de escleroterapia com espuma de polidocanol guiada por ultrassom no período de janeiro de 2013 a dezembro de 2014. Foram analisados tempo de cicatrização da úlcera, melhora de sintomas clínicos, recanalização das veias tratadas, recidiva dos sintomas e da úlcera venosa.

**Resultados:**

Foram analisados 15 pacientes do sexo feminino (78,9%) e quatro do sexo masculino (21,1%). A média geral de idade foi de 53 anos. O tempo de seguimento dos pacientes variou de 448 dias a 1.276 dias (média de 791 dias). O tempo médio de presença das úlceras foi de 53 meses. Na avaliação pós-procedimento, foram observadas recanalização total em 15,7%, recanalização parcial em 21% e oclusão em 47,3% das veias tratadas. Apenas em um caso foi observada recidiva da úlcera. Pela avaliação das médias do *Venous Clinical Severity Score* (VCSS), houve diferença significativa antes e após o procedimento, com variação entre os grupos de 11,2 (p < 0,01).

**Conclusões:**

A escleroterapia por espuma guiada por ultrassom apresenta altas taxas de sucesso terapêutico, com índices de cicatrização de úlceras venosas elevados.

## INTRODUÇÃO

As veias varicosas são manifestações resultantes da insuficiência venosa crônica (IVC) decorrente da hipertensão venosa de longa duração^1^^,^ 2. Ocorre em 25% das mulheres e 15% dos homens em idade produtiva^1^, trazendo um grande impacto socioeconômico e também na qualidade de vida desses indivíduos.

Estudos brasileiros encontraram uma prevalência da IVC de 35,5% da população^3^, sendo que a estimativa de prevalência de úlcera venosa varia entre 0,2 e 1,0%^4^. Outros estudos também publicados no país, por sua vez, verificaram a prevalência de sinais avançados relacionados a varizes e encontraram uma média de 19,7% para edema, 5,7% para hiperpigmentação, 1,4% para eczema e 0,6% para dermatofibrose^5^.

Desde 1994, a classificação das doenças venosas, baseada em dados clínicos (C), etiologia (E), distribuição anatômica (A) e a fisiopatologia (P), denominada classificação CEAP^6^, vem sendo utilizada globalmente, com algumas modificações realizadas em 2004 para aprimorá-la^7^. A avaliação clínica por si só não estabelece os níveis anatômicos envolvidos, sendo necessário o uso de exames complementares^8^. Dentre os métodos não invasivos, o mais utilizado é a ultrassonografia Doppler colorida, método indolor que pode ser aplicado quantas vezes forem necessárias, com a vantagem de confirmar o diagnóstico tanto pela avaliação do diâmetro das veias como pela presença de refluxo ou oclusões. É considerado um dos métodos para determinação acurada da distribuição e extensão da doença venosa^9^.

A escleroterapia com espuma de polidocanol, um dos tratamentos usados na IVC, possui a vantagem de: ser um procedimento minimamente invasivo e de fácil execução, podendo ser feita a nível ambulatorial e proporcionando ao paciente o retorno domiciliar precoce e uma retomada de atividades cotidianas. Não existem limitações de execução técnica em recidivas, visto que, em casos de recanalização, os mesmos pacientes podem ser submetidos novamente ao método^10^. O polidocanol, agente comumente usado nesse tipo de terapia na forma de espuma, é indolor e possui uma baixa incidência de reações alérgicas. O sucesso terapêutico se evidenciou no estudo de Gonzalez-Zeh et al.^11^, o qual demonstrou que após 1 ano, houve sucesso no procedimento de ablação em 77% dos indivíduos.

A técnica atual é baseada nos métodos de Tessari et al.^12^, em que uma mistura de líquido esclerosante e ar forma uma espuma através de agitação utilizando duas seringas conectadas entre si por uma torneira de três vias. A injeção de espuma pode ser auxiliada por ultrassom com Doppler. Este contribui para guiar a punção e observar, durante o procedimento, a progressão da espuma pelo segmento venoso a ser tratado^13^^,^ 14.

O objetivo do presente estudo é relatar o desfecho e a evolução a curto e médio prazo de pacientes com IVC de membros inferiores com úlcera tratados com escleroterapia com espuma de polidocanol guiada por ultrassom.

## MÉTODOS

Estudo descritivo de pacientes com IVC CEAP C6 submetidos ao tratamento de espuma com polidocanol guiada por ultrassom no Serviço de Cirurgia Vascular de um hospital-escola no período de janeiro de 2013 a dezembro de 2014. Os dados do período do tratamento inicial foram coletados com base em um banco de dados existente no serviço. Através de entrevista pré-estruturada, os pacientes foram reavaliados em nova consulta no período de setembro de 2015 a dezembro de 2015. Foram incluídos no estudo todos os pacientes com faixa etária de 18 a 80 anos de idade, portadores de úlcera venosa e submetidos a escleroterapia com espuma de polidocanol guiada por ultrassom. Foram excluídos pacientes gestantes, menores de 18 anos, maiores de 80 anos, com antecedente de trombose venosa profunda recente ou tardia não recanalizada, ou com doença arterial crônica e doença arterial aguda.

Através da pesquisa do banco de dados, foram recolhidos parâmetros como: nome do paciente, idade, sexo, registro do prontuário, membro afetado, comorbidades, veia tratada e data do procedimento. Além disso, foram coletadas informações do exame de Doppler anterior ao tratamento, analisando assim o refluxo no sistema venoso profundo e superficial.

Na reavaliação dos pacientes, foi realizado novo exame físico, coleta de informações complementares relacionadas com a cicatrização da úlcera e com os procedimentos realizados e foi observado, ainda, se houve ou não recidiva da úlcera. Nesse mesmo momento, foi realizada uma nova ultrassonografia com Doppler para avaliar, através de um profissional treinado, as veias tratadas previamente. Os parâmetros analisados foram a permanência da oclusão ou a recanalização total ou parcial da veia tratada.

Parâmetros do escore internacional *Venous Clinical Severity Score* (VCSS) foram incluídos na análise antes e depois da escleroterapia. Por meio desse escore, avalia-se a gravidade da IVC através dos parâmetros numéricos relacionados com dor, varizes, edema venoso, inflamação, enduração, número de úlceras, duração da úlcera, tamanho maior da úlcera, terapia compressiva e pigmentação da pele^15^.

As variáveis categóricas foram submetidas a análise estatística descritiva com média e mediana. O tempo de cicatrização foi calculado a partir da data da primeira sessão do tratamento. O tempo de recorrência foi calculado a partir da data de cicatrização da úlcera. Para a análise dos valores de VCSS pré e pós-tratamento, foi utilizado o teste paramétrico *t* de Student para amostras pareadas.

A realização deste estudo foi aprovada pelo Comitê de Ética e Pesquisa da instituição e pela Plataforma Brasil sob número de parecer 1.111.919.

## RESULTADOS

No período analisado, 73 pacientes com úlcera venosa foram tratados. Foi realizada a reavaliação clínica de 19 pacientes (26%), após convocação por contato telefônico, com tempo de seguimento variando de 448 dias a 1.276 dias (média de 791 dias). Houve dificuldade no recrutamento da maior parte dos pacientes, principalmente devido ao fato de residirem em cidades distantes do serviço de origem (52 pacientes), tornando a amostra analisada menor que o número total tratado.

Dos 19 pacientes que realizaram a reavaliação, 15 eram do sexo feminino (78,9%) e 4 eram do sexo masculino (21,1%). A média geral de idade foi de 63,6 anos. Nove pacientes apresentavam comorbidades associadas (47,3%). A mais prevalente foi hipertensão arterial sistêmica, presente em seis dos 19 pacientes (31,5%), seguida de diabetes melito, presente em dois dos 19 pacientes (10,5%), e cirrose hepática, presente em um paciente (5,3%).

O membro mais acometido pela úlcera venosa foi o membro inferior esquerdo, afetado em 10 dos 19 pacientes avaliados (52,6%). O tempo médio de atividade das úlceras foi de 53 meses.

**Figura 1:**
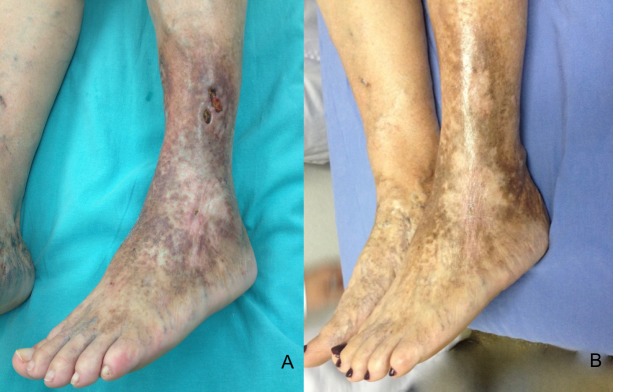
(A) Úlcera venosa ativa em face lateral de perna esquerda; (B) Pós-tratamento com escleroterapia com espuma de polidocanol guiada por ultrassom, com tempo de cicatrização de 30 dias.

**Figura 2:**
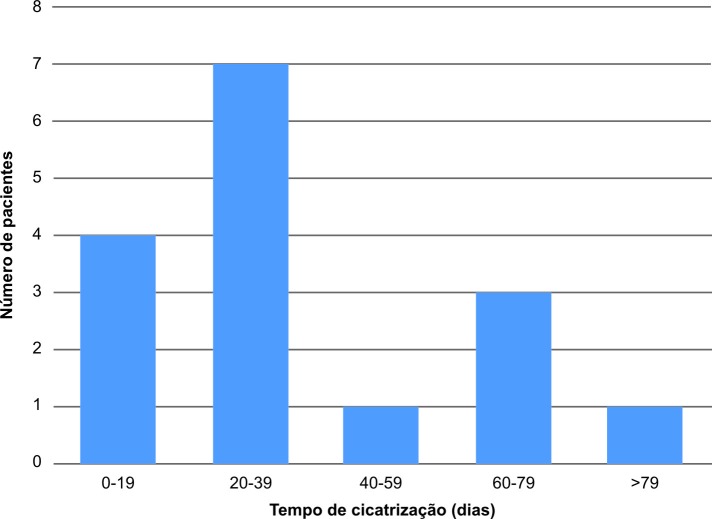
Distribuição do período (em dias) da cicatrização da úlcera após o procedimento.

Treze dos 19 pacientes (68,42%) passaram por apenas uma aplicação, e seis dos 19 (31,5%) passaram por mais de uma aplicação da espuma, com taxa de cicatrização de 89% (Figuras 1A e 1B), em um tempo médio de 31,94 dias (Figura 2).

O padrão de refluxo venoso pré-tratamento nos membros tratados foi encontrado com distribuição irregular entre sistemas venosos profundo, superficial e perfuro-comunicantes. Apenas um paciente apresentou refluxo em sistema venoso profundo (5,2% dos membros). O maior número dos casos (12 pacientes) apresentou refluxo de veia safena magna em toda sua extensão. Um paciente apresentava refluxo segmentar em veia safena magna apenas em perna. A veia safena parva foi considerada incompetente em quatro membros, sendo que em um deles houve a associação de refluxo em veia safena magna. Dois pacientes (10% dos casos) apresentavam apenas refluxo de sistema pérfuro-comunicante.

Na avaliação após o procedimento, foi observada recanalização total de 21%; recanalização parcial de 26% e oclusão de 53%. Porém, só houve recidiva de úlcera em apenas um caso (5%), com 728 dias após o tratamento inicial. Esse caso apresentava recanalização total da veia safena magna tratada previamente.

**Figura 3:**
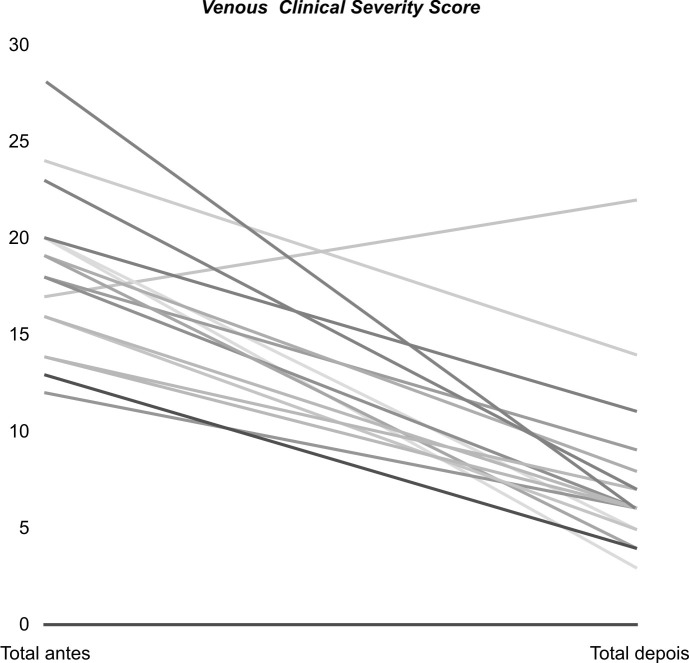
Distribuição dos valores do *Venous Clinical Severity Score* (VCSS) dos pacientes antes e após a escleroterapia com espuma de polidocanol guiada por ultrassom.

Antes do tratamento os valores de VCSS encontrados variaram de 12 a 28 (média de 18,7). Após o tratamento, os novos valores variaram entre 3 e 22 (média de 7,5). Apenas em um caso observou-se piora clínica, com aumento de 17 para 22. Houve alteração significativa dos valores da VCSS antes e após o procedimento (p < 0,01), com média de 11,2. Utilizando como intervalo de confiança de 95%, os valores variaram em números absolutos de 8,2 a 14,1 (Figura 3).

## DISCUSSÃO

A escleroterapia é alternativa eficaz e atraente para o tratamento da insuficiência venosa crônica com refluxo observado em sistema superficial e pérfuro-comunicante. Pode ser utilizada facilmente em regimes ambulatoriais, principalmente a escleroterapia com espuma.

O tempo de acompanhamento desta amostra é superior a vários trabalhos já publicados. Com período de 448 dias a 1.276 dias (média de 791 dias), foi possível analisar a longo prazo o efeito do tratamento esclerosante no grupo específico de pacientes com úlcera, enfatizando a ocorrência de recidivas e recanalizações das veias tratadas.

O perfil epidemiológico dos pacientes analisados neste estudo é heterogêneo. Trata-se de uma amostra pequena, mas que mantém o padrão dos outros relatos já publicados, que engloba pacientes de várias faixas etárias e com múltiplas comorbidades^10^^,^ 15.

Em um estudo para a avaliação dos resultados imediatos a fim de avaliar a eficácia do procedimento, provou-se que uma única sessão foi suficiente para tratar 58% dos pacientes portadores de IVC. A eliminação completa das veias varicosas ocorreu em 87% dos indivíduos^16^, indicando sucesso terapêutico elevado. Esse grupo não pode ser comparado ao do presente estudo, visto que o desfecho analisado no primeiro estudo foi a eliminação das veias varicosas, e no atual estudo foi a oclusão da veia ou cicatrização da úlcera.

Em relação aos portadores de IVC grave, Silva et al^17^. mostraram que a escleroterapia por espuma guiada por ultrassom possui um efeito significativo sobre os pacientes. Houve cicatrização da úlcera em 84,2% dos casos, com um tempo médio de 37 dias. A recanalização ocorreu em uma porcentagem de 31,5%, porém associado a sintomas e reaparecimento da úlcera foi de 11,8%.

O presente estudo demonstrou índices de cicatrização das úlceras semelhantes aos relatados na literatura. O índice de 89% (em 791 dias) é semelhante ao estudo de Howard et al., em que se observou um índice de cicatrização de úlcera de 86% em 12 meses, mostrando a eficácia da escleroterapia por espuma a longo prazo em estágios C6 da classificação CEAP de IVC^18^. A recorrência da úlcera venosa no atual estudo foi observada em 5,2%. Segundo Grover et al., em 50 membros, com uma mediana de acompanhamento de 15,2 meses, houve uma recorrência de 4/50 (8%) em 12 meses^19^.

Foi observado no presente estudo taxas de recanalizações parciais e totais pós- tratamento de 26% e 21%, respectivamente. Em alguns aspectos, esses resultados são semelhantes aos relatados por Howard et al.^18^, que observaram uma completa recanalização em 27% e recanalização parcial em 58%. Wiliamsson et al^20^. observaram, após um ano, através do ultrassom com Doppler, 86% (25/29) das veias examinados ocluídas, uma parcialmente ocluída e três recanalizadas. Esses valores são diferentes dos que encontramos no presente estudo. Vários fatores influenciam as taxas de recanalização, como os diâmetros das veias tratadas, por exemplo. Porém, essa informação não foi estudada neste momento.

O VCSS foi outro critério analisado pré e pós procedimento. Masuda et al^21^. notaram melhora significativa nos escores de 37 pacientes com úlcera ativa tratados com espuma de polidocanol. Apenas um dos pacientes do atual estudo obteve pontuação maior no pós-procedimento, indicando piora. Trata-se de paciente que mantém úlcera ativa, mesmo com o uso de compressão elástica pós-tratamento.

Apesar do tamanho pequeno da amostra, foi possível analisar algumas variáveis importantes para o real entendimento desse tipo de tratamento utilizado na IVC. Há projeção para manter acompanhamento desses pacientes, com a inclusão de novos casos, para programação de nova análise.

## CONCLUSÃO

A escleroterapia por espuma guiada por ultrassom apresenta altas taxas de sucesso terapêutico, além de índices de cicatrização de úlceras venosas elevados e duradouros a curto e médio prazo. Ocorreu recanalização das veias tratadas em número expressivo; porém, na maior parte dos casos esse fato não gera piora na gravidade da doença.
