# Supplementary material for: Impacto da escleroterapia com espuma de polidocanol guiada por ultrassom em pacientes com úlcera venosa
Source: J Vasc Bras. 2017 Jul-Sep;16(3):239–43. [Article in Portuguese] doi: 10.1590/1677-5449.002717 (PMC5868941; doi:10.1590/1677-5449.002717)
Supplement: Translation

# Translation: Impact of ultrasound-guided polidocanol foam sclerotherapy in patients with venous ulcers

**Authors:** Melissa Andreia de Moraes Silva, Álefy Zanelato Pereira Araujo, Jéssica Funchal do Amaral, Seleno Glauber de Jesus-Silva, Rodolfo Souza Cardoso, Fausto Miranda

**Affiliations:** 1 Faculdade de Medicina de Itajubá – FMIt, Cirurgia Vascular, Itajubá, MG, Brazil.; 2 Universidade Federal de São Paulo – UNIFESP, Escola Paulista de Medicina, Cirurgia Vascular, São Paulo, SP, Brazil.

**Keywords:** sclerotherapy, varicose ulcer, healing, relapse

## Abstract

**Background:**

Ultrasound-guided polidocanol foam sclerotherapy is used to treat patients with venous ulcers. It is a minimally invasive procedure and is simple to perform, but it has high relapse rates.

**Objectives:**

To report short to medium term results in patients with venous ulcers treated using ultrasound-guided polidocanol foam sclerotherapy.

**Methods:**

A sample of 19 patients who had been treated with ultrasound-guided polidocanol foam sclerotherapy between January 2013 and December 2014 were followed-up. Time taken for ulcers to heal, improvement of clinical symptoms, recanalization of treated veins, and relapse of symptoms and of venous ulcers were analyzed.

**Results:**

Fifteen of the patients analyzed were female (78.9%) and four were male (21.1%). Overall mean age was 53 years. Follow-up times ranged from 448 days to 1,276 days (mean of 791 days). The mean duration of active ulcers was 53 months. At postoperative follow-up assessments, total recanalization was observed in 15.7%, partial recanalization in 21%, and occlusion in 47.3% of the veins that had been treated. There was only one case of ulcer relapse. Analysis of mean Venous Clinical Severity Scores (VCSS) revealed a significant difference from before to after the procedure, with a variation of 11.2 (p < 0.01).

**Conclusions:**

Ultrasound-guided foam sclerotherapy has high rates of therapeutic success and achieves high rates of venous ulcer healing.

## INTRODUCTION

Varicose veins are manifestations of chronic venous insufficiency (CVI) resulting from long duration venous hypertension.^1^^,^ 2 They affect 25% of women and 15% of men of working age,^1^ have a major socioeconomic impact, and are highly detrimental to patients’ quality of life.

Brazilian studies have reported a CVI prevalence of 35.5% of the population,^3^ while estimates of the prevalence of venous ulcers vary from 0.2 to 1.0%.^4^ In turn, other studies published in the country have investigated the prevalence of advanced signs related to varicose veins, finding mean rates of 19.7% for edema, 5.7% for hyperpigmentation, 1.4% for eczema, and 0.6% for dermatofibrosis.^5^

Since 1994, a classification of venous diseases based on clinical data (C), etiology (E), anatomic distribution (A) and pathophysiology (P), known as the CEAP classification,^6^ has been adopted worldwide, although certain modifications were introduced in 2004 to improve it.^7^ Clinical assessment alone does not determine the anatomic levels involved, for which additional tests and examinations are needed.^8^ The most widely used noninvasive method is color Doppler ultrasonography, which is a painless method that can be employed as often as necessary, offering the advantage of confirming diagnosis both by evaluation of the diameter of veins and by identification of the presence of reflux or occlusions. It is one of the available methods for accurate determination of the distribution and extent of venous disease.^9^

Sclerotherapy with polidocanol foam, one of the methods used to treat CVI, offers the advantages of being a minimally invasive and easily executed procedure that can be provided in outpatients settings and which enables patients to return home and resume daily activities early. There are no limitations to using the technique after relapses, since in cases of recanalization the same patients can be treated again with the same method.^10^ Polidocanol, in the form of foam, is the agent generally used in this type of treatment and it is painless and has a low incidence of allergic reactions. Treatment success rates have been reported by Gonzalez-Zeh et al.,^11^ who showed that after 1 year the procedure had achieved successful ablation in 77% of those treated.

The current technique is based on methods described by Tessari et al.,^12^ in which a sclerosing agent and air are mixed together to form foam using two syringes and a three-way stopcock. Foam injection can be facilitated using Doppler ultrasound to guide the puncture and to observe the foam progressing through the venous segment as the procedure is performed.^13^^,^ 14

The objective of the present study is to describe the short and medium term outcomes of patients with lower limb CVI with ulcers who were treated with ultrasound-guided polidocanol foam sclerotherapy.

## METHODS

This is a descriptive study of patients with CEAP C6 grade CVI who were treated with ultrasound-guided polidocanol foam by the vascular surgery service of a teaching hospital between January 2013 and December 2014. Data from the initial treatments were acquired from a database maintained by the service. The patients were reassessed at consultations held from September 2015 to December 2015, using structured interviews. The study enrolled all patients within the age range of 18 to 80 years of age who had had venous ulcers and had been treated with ultrasound-guided polidocanol foam sclerotherapy. Patients were excluded if they were pregnant, under the age of 18 or over the age of 80, had a recent or early history of deep venous thrombosis that had not been recanalized, or had chronic or acute arterial disease.

The database was searched for information such as: patient name, age, sex, medical record, affected limb, comorbidities, vein treated, and date of procedure. Additionally, information was acquired from the Doppler examination conducted prior to treatment, covering reflux in the deep and superficial vein systems.

At the reassessment consultation, patients underwent another physical examination, supplementary information related to ulcer healing and the procedures conducted was collected, and observations were made on whether or not ulcer relapse had occurred. At the same consultation, a trained professional used Doppler ultrasonography to assess veins that had been treated. The findings were recorded as maintenance of occlusion, total recanalization, or partial recanalization.

Parameters needed to calculate the international Venous Clinical Severity Score (VCSS) were included in the before and after sclerotherapy analysis. This score evaluates the severity of CVI in terms of numerical parameters for pain, varicose veins, venous edema, inflammation, hardening, number of ulcers, duration of ulcer, size of largest ulcer, compression therapy, and skin pigmentation.^15^

Categorical variables were analyzed using descriptive statistics, with calculation of means and medians. Time taken for healing was calculated from the date of the first treatment session. Time before relapse was calculated from the date of ulcer healing. Student’s parametric *t* test for paired samples was used to analyze VCSS results for before and after treatment.

This study was approved by the institutional Research Ethics Committee and the Plataforma Brasil, under ruling number 1.111.919.

## RESULTS

A total of 73 patients with venous ulcers were treated during the period analyzed. Of these, 19 patients (26%) attended for clinical reassessment, after being invited by telephone, with follow-up times ranging from 448 days to 1,276 days (mean of 791 days). The major difficulty with recruiting patients originally treated was caused by the fact that the majority (52 patients) live in towns that are a long way from the treating service, with the result that the sample reassessed was smaller than the total cohort of patients treated.

Of these 19 patients who presented for reassessment, 15 were female (78.9%) and 4 were male (21.1%). The overall mean age was 63.6 years. Nine patients had associated comorbidities (47.3%). The most prevalent of these was systemic arterial hypertension, in six of the 19 patients (31.5%), followed by diabetes mellitus, in two of the 19 patients (10.5%), and cirrhosis of the liver, in one patient (5.3%).

The limb most frequently affected by venous ulcers was the left lower limb, in 10 of the 19 patients (52.6%). The mean duration of ulcer activity was 53 months.

Thirteen of the 19 patients (68.4%) were treated in a single session, while six of the 19 (31.5%) patients were given more than one administration of foam, the proportion of ulcer healing was 89% (Figures 1A and 1B), and mean time taken for healing was 31.94 days (Figure 2).

**Figure 1:** (A) Active venous ulcer on lateral surface of left leg; (B) After treatment with ultrasound-guided polidocanol foam sclerotherapy, with healing time of 30 days.

**Figure 2:** Distribution of time (in days) taken for ulcers to heal after the procedure.

The pattern of venous reflux observed prior to treatment in the limbs to be treated was irregularly distributed between deep, superficial, and perforating-communicating venous systems. Just one patient had reflux in the deep vein system (5.2% of limbs). The greater proportion of cases (12 patients) exhibited reflux along the entire extension of the great saphenous vein. One patient had segmental reflux in the great saphenous vein in one leg only. The small saphenous vein was considered incompetent in four limbs, in one of which there was also reflux in the great saphenous vein. Two patients (10% of cases) only had reflux in the perforating-communicating system.

The reassessment after the procedure detected total recanalization in 21%; partial recanalization in 26%, and maintenance of occlusion in 53%. However, ulcer relapse only occurred in one case (5%), 728 days after the initial treatment. This case had total recanalization of the great saphenous vein that had been treated.

Before treatment, the VCSS scores ranged from 12 to 28 (mean of 18.7). After treatment, the scores varied from 3 to 22 (mean of 7.5). Clinical deterioration was only observed in one case, with the VCSS score increasing from 17 to 22. There was a significant change in VCSS scores from before to after the procedure (p < 0.01), with a mean difference of 11.2. Using a 95% confidence interval, the absolute values ranged from 8.2 to 14.1 (Figure 3).

**Figure 3:** Distribution of Venous Clinical Severity Score (VCSS) results for patients before and after ultrasound-guided polidocanol foam sclerotherapy.

## DISCUSSION

Sclerotherapy is an effective and attractive option for treatment of chronic venous insufficiency with reflux observed in both superficial and perforating-communicating systems. It can be easily performed in outpatients settings, and this is particularly true of foam sclerotherapy.

The follow-up time reported in the present study is greater than in many published studies. With follow-up times ranging from 448 days to 1,276 days (mean of 791 days), it was possible to analyze the long-term effects of sclerosing treatment in a specific group of patients with ulcers, focusing on relapses and recanalization of the veins treated.

The patients analyzed in this study have a heterogeneous epidemiological profile. It is a small sample, but it conforms to the pattern reported in previously published work, encompassing patients of varying ages and with multiple comorbidities.^10^^,^ 15

In a study conducted to evaluate immediate results in order to assess the efficacy of the procedure, it was shown that a single session was sufficient to treat 58% of patients with CVI. Total elimination of varicose veins was achieved in 87% of the sample,^16^ indicating a high therapeutic success rate. However, that sample cannot be compared to the patients in the present study, since the outcome analyzed was elimination of varicose veins, whereas in the present study it was occlusion of the vein or healing of the ulcer.

With relation to patients with severe CVI, Silva et al.^17^ showed that ultrasound-guided foam sclerotherapy had a significant effect on patients. Ulcers healed in 84.2% of cases, with a mean healing time of 37 days. Recanalization was observed in 31.5%, but the rate of recanalization with symptoms and recurrence of ulcers was 11.8%.

The present study observed rates of ulcer healing similar to those reported in the literature. The rate of 89% (at 791 days) is similar to that seen in a study by Howard et al., who observed an ulcer healing rate of 86% at 12 months, showing the long-term efficacy of foam sclerotherapy at grade C6 of the CEAP CVI classification.^18^ The venous ulcer recurrence rate in the present study was 5.2%. According to Grover et al., in a sample of 50 limbs with a median follow-up time of 15.2 months, there was recurrence in 4/50 (8%) at 12 months.^19^

The partial and total recanalization rates observed in the present study were 26% and 21%, respectively. In some aspects these results are similar to those reported by Howard et al.,^18^ who observed complete recanalization in 27% and partial recanalization in 58%. One year after treatment, Wiliamsson et al.^20^ observed that 86% (25/29) of veins examined with Doppler ultrasound were occluded, one was partially occluded, and three were recanalized. These values are different to the findings of the current study. Several different factors influence recanalization rates, such as the diameters of the veins treated, for example. However, this information was not analyzed in this study.

The VCSS score was another criterion analyzed before and after the procedure. Masuda et al.^21^ noted significant improvements in the scores for 37 patients with active ulcers after treatment with polidocanol foam. Just one of the patients in the present study had a higher score after the procedure, indicating deterioration. This was a patient whose ulcer remained active, despite wearing elastic compression after treatment.

Despite the small sample size, it was possible to analyze certain variables that are important to a real understanding of this type of treatment for CVI. The plan is to continue following these patients, and include additional cases, to enable another analysis in the future.

## CONCLUSIONS

Ultrasound-guided foam sclerotherapy achieved high treatment success rates and high and sustained rates of venous ulcer healing over the short and medium term. There was a considerable proportion of recanalization of treated veins; but in the majority of cases this did not cause disease severity to exacerbate.
